# A phase I first-in-man study to investigate the pharmacokinetics and safety of liposomal dexamethasone in patients with progressive multiple myeloma

**DOI:** 10.1007/s13346-022-01268-6

**Published:** 2023-01-02

**Authors:** Josbert Metselaar, Twan Lammers, Amelie Boquoi, Roland Fenk, Fabio Testaquadra, Mirle Schemionek, Fabian Kiessling, Susanne Isfort, Stefan Wilop, Martina Crysandt

**Affiliations:** 1 Institute for Experimental Molecular Imaging, Medical Faculty, RWTH Aachen University, Aachen, Germany; 2 Department of Hematology, Oncology, Hemostaseology and Stem Cell Transplantation, Medical Faculty, RWTH Aachen University, Aachen, Germany; 3 Center for Integrated Oncology, Aachen Bonn Cologne Duesseldorf (CIO ABCD), Aachen, Germany; 4 Department of Hematology, Oncology and Clinical Immunology, University Hospital Duesseldorf, Heinrich-Heine University, Duesseldorf, Germany

**Keywords:** Multiple myeloma, Targeted drug delivery, Pegylated liposomes, Dexamethasone

## Abstract

Despite the introduction of multiple new drugs and combination therapies, conventional dexamethasone remains a cornerstone in the treatment of multiple myeloma (MM). Its application is, however, limited by frequent adverse effects of which the increased infection rate may have the strongest clinical impact. The efficacy-safety ratio of dexamethasone in MM may be increased by encapsulation in long-circulating PEG-liposomes, thereby both enhancing drug delivery to MM lesions and reducing systemic corticosteroid exposure. We evaluated the preliminary safety and feasibility of a single intravenous (i.v.) infusion of pegylated liposomal dexamethasone phosphate (Dex-PL) in heavily pretreated relapsing or progressive symptomatic MM patients within a phase I open-label non-comparative interventional trial at two dose levels. In the 7 patients that were enrolled (prior to having to close the study prematurely due to slow recruitment), Dex-PL was found to be well tolerated and, as compared to conventional dexamethasone, no new or unexpected adverse events were detected. Pharmacokinetic analysis showed high and persisting concentrations of dexamethasone in the circulation for over a week after i.v. administration, likely caused by the long-circulation half-life of the liposomes that retain dexamethasone as the inactive phosphate prodrug form, something which could significantly limit systemic exposure to the active parent drug. Thus, despite the limitations of this small first-in-man trial, Dex-PL seems safe and well tolerated without severe side effects. Follow-up studies are needed to confirm this in a larger patient cohort and to evaluate if i.v. Dex-PL can provide a safer and more efficacious dexamethasone treatment option for MM.

**Graphical Abstract:**

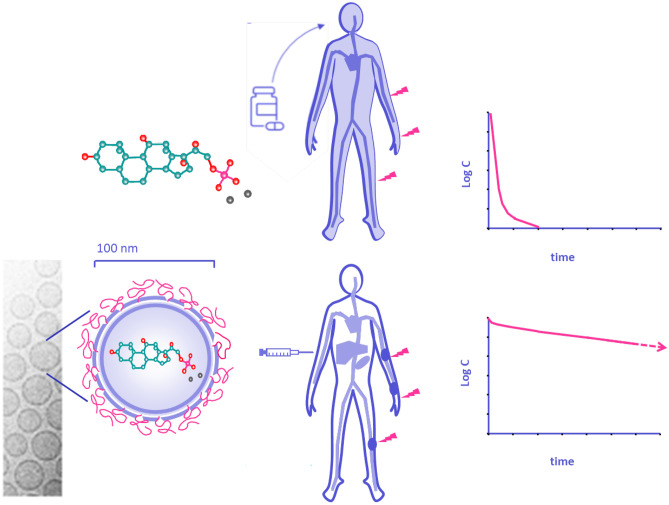

**Supplementary Information:**

The online version contains supplementary material available at 10.1007/s13346-022-01268-6.

## Introduction

Multiple myeloma (MM) is a plasma cell malignancy that is characterized by the accumulation of clonal plasma cells, mainly in the bone marrow, leading to clinical manifestations such as anemia, bone destruction, and renal insufficiency [1]. Despite progress in our understanding of disease’s biology and the improvement in therapeutic options, therapy remains palliative [2, 3]. Almost all patients with multiple myeloma (MM), who respond to initial treatment, will eventually relapse and require further therapy [4]. Standard therapy comprises a combination of high dose chemotherapy, local radiation therapy, and, in the younger and fitter patients, autologous stem cell transplantation. Proteasome inhibitors, immunomodulatory drugs, monoclonal antibodies, corticosteroids, and alkylating agents are the most active agents [5]. Additionally, a variety of new drugs and drug classes and strategies (e.g., CAR T cell therapy) have changed the landscape significantly [6, 7].

Corticosteroids, and in particular dexamethasone, are an important mainstay in the treatment of MM [8–10]. In the last decades due to the previously rather limited number of alternative treatment options, dexamethasone was quite often used as a monotherapy for MM. A high dose oral treatment regimen of up to 160 mg per week divided over 4 daily doses has been popular albeit at the cost of side effects, including psychiatric, metabolic and diabetes-related complications, gastrointestinal disorders, hypertension, and especially life-threatening infections [11]. Considering the unfavorable side effect profile and the lower efficacy compared to the newer targeted treatment options available, dexamethasone monotherapy is nowadays rarely given. Rather, the drug is co-administered with the majority of current MM combination therapies. The standard dexamethasone dosage in combination settings involves a lower oral dose of weekly 20–40 mg [12], although doses of 320 mg within 4 weeks are still recommended in some treatment regimens [13]. Even though a number of new drugs have been approved for the treatment of MM especially in the last decade (such as daratumumab, carfilzomib, and pomalidomide), dexamethasone remains the backbone of almost every combination regimen [7].

Dexamethasone exerts its beneficial effect in MM in several ways. Besides acting on plasma cells directly at the level of growth inhibition and induction of apoptosis [14, 15], it also reduces the production of tumor-promoting interleukins (most notably IL-6) by osteoclasts and macrophages in the bone marrow, where plasma cells home and form the tumor lesion [16]. While it is obvious that these bone marrow lesions are the target site for agents like dexamethasone, a small amount of drug actually fully infiltrates these sites upon systemic administration, and high levels accumulate off-site in healthy tissues due to suboptimal pharmacokinetic properties of glucocorticoids, particularly the large volume of distribution and rapid clearance [17].

**Fig. 1:**
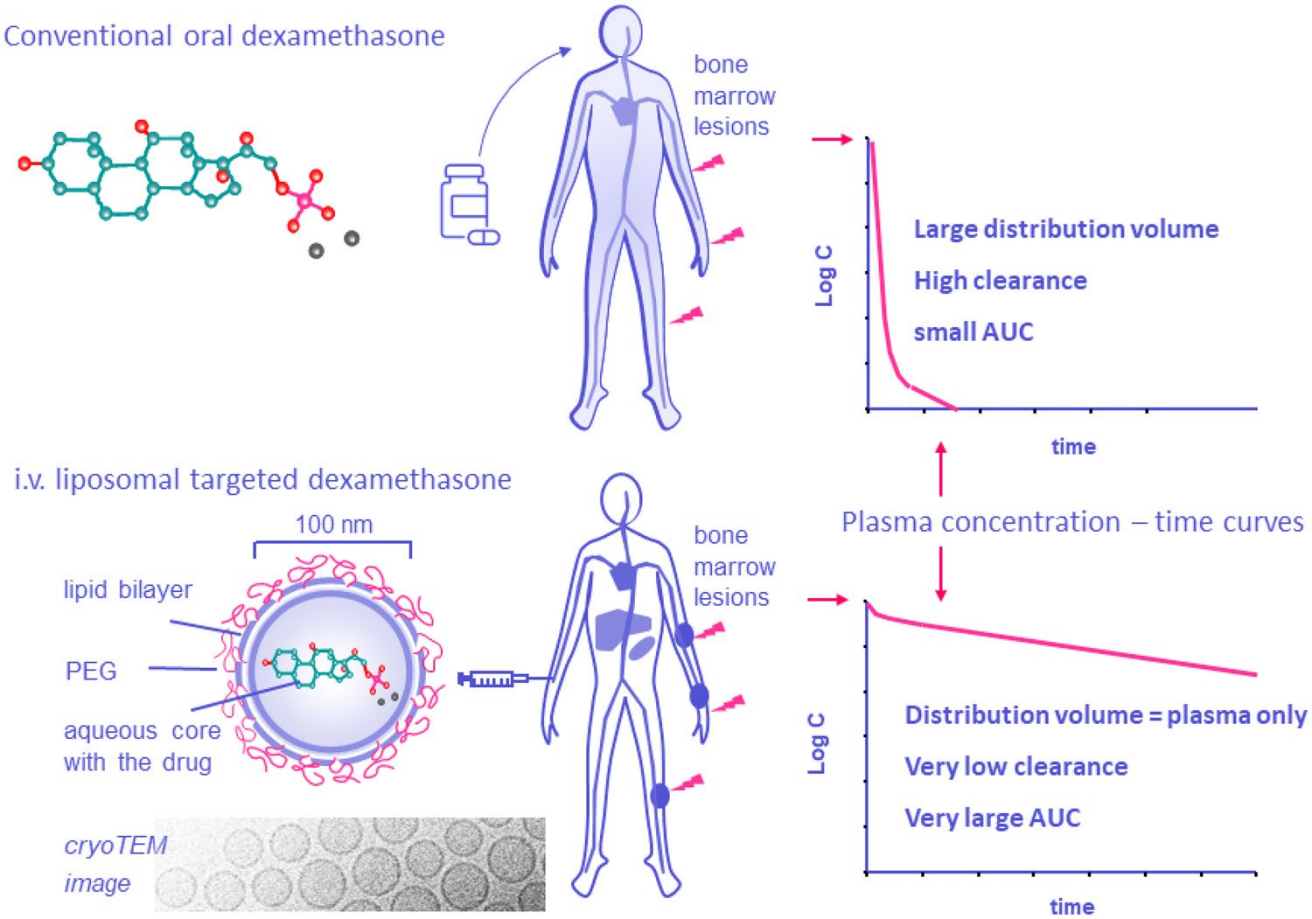
Schematic representation of the difference between conventional oral dexamethasone treatment and intravenously infused PEG-liposomal dexamethasone, emphasizing particularly the striking difference in PK profiles and target site accumulation obtained after liposomal treatment [24]

We postulate that the therapeutic value of dexamethasone in MM may be increased by utilizing the concept of nanomedicine-based drug delivery to MM bone marrow lesions. A graphical comparison of the advantage of this concept in comparison with free dexamethasone administration is given in Fig. 1. Presented in this figure is the most obvious drug delivery formulation for this purpose, namely, the pegylated liposome, which is a small-sized (100 nm) phospholipid bilayer vesicle enriched with cholesterol and coated with a layer of short-chained poly(ethylene glycol) (PEG). Cholesterol and PEG prevent premature removal of the phospholipid vesicles by the mononuclear phagocyte system (MPS) and impart so-called “long-circulating behavior” after i.v. infusion [18]. PEG-liposomes have three well-established properties that render them valuable in the treatment of MM: (1) a natural propensity for the bone marrow (besides liver and spleen) [19], which is also the site where plasma cells localize, accumulate and build their own supportive microenvironment; (2) a proven ability to target tumors in general, making use of enhanced vascular permeability as a result of neovascularization and inflammation [20, 21]; and (3) after extravasation at pathological sites, PEG-liposomes show strong uptake by macrophages, a cell type which has gained a reputation as key regulator of MM progression [22, 23].

An important advantage of using PEG-liposomes over other drug delivery systems is that they are well established in the clinic, with proven therapeutic value and a fully investigated safety profile. Several products based on PEG-liposomes are clinically used or under investigation [24]. The therapeutic value of PEG-liposomes is more specifically validated in MM by the successful addition of PEG-liposomal doxorubicin to the chemotherapeutic arsenal in relapsed or refractory MM, producing an increase in progression-free survival [25].

We here report the results of an exploratory phase 1 clinical study (ClinicalTrials.gov Identifier NCT03033316) with the primary aim of evaluating safety and pharmacokinetics and secondary aim of evaluating the efficacy of the abovementioned PEG-liposomal dexamethasone (Dex-PL) formulation in patients with heavily pretreated, symptomatic MM.

## Methods

### Study design

We conducted an 8-week open-label, multi-center, dose-escalating phase I study in patients with pretreated progressive MM. The study was registered under EUdraCT number 2014–005137-32 and approved by both the Independent Ethics Committee at the Medical Faculty of RWTH Aachen University and the Federal Institute for Drugs and Medical Devices (BfArM) and conducted in accordance with the ethical principles of ICH’s Good Clinical Practice guidelines and the Declaration of Helsinki (https://database.ich.org/sites/default/files/ICH_E6-R3_GCP-Principles_Draft_2021_0419.pdf). Written informed consent for study participation was obtained from each patient prior to screening and the performance of any study-specific procedures.

### Patient inclusion criteria

All patients older than 18 years old with a relapse or a progression of previously diagnosed symptomatic MM according to International Myeloma Working Group (IMWG) criteria and who were previously treated with at least two lines of therapy including at least one proteasome inhibitor and at least one of the immunomodulatory imide drugs (IMiDs) were included. Patients were to have a measurable disease (M-protein and/or free light chains) in serum and/or urine.

### Patient exclusion criteria

Key exclusion criteria included the following: previously documented irresponsiveness to dexamethasone monotherapy, a diagnosis of plasma cell leukemia, detectable signs of hepatitis infection, other infections requiring systemic treatment, and treatment with oral or injectable (including intra-articular) corticosteroids (CS) within 4 weeks prior to the screening visit.

### Liposomal dexamethasone study drug

Pegylated liposomal dexamethasone (Dex-PL) was provided by Enceladus Pharmaceuticals, Naarden, the Netherlands. Dex-PL is composed of dipalmitoyl phosphatidyl choline, pegylated distearoyl phosphatidyl ethanolamine, and cholesterol, which constitute the lipid bilayer of carefully sized 100 nm vesicles that stably encapsulate dexamethasone sodium phosphate in the aqueous interior. Before treatment, the required dose of this composition was diluted in 0.9% sodium chloride solution and administered intravenously during 1 to 2 h depending on the dose, using a stepwise protocol of increased infusion rates so as to minimize the chance of infusion-related side effects.

### Study procedures

The design of the study was based on the standard “3 + 3”-algorithm as described by Storer [26]. In this setting, 3 patients per dose level were to be enrolled. If one patient reached a dose limiting toxicity (DLT), another 3 patients would be enrolled in the same dose level. DLT was defined as any toxicity of grade 3 or higher and judged to be related to the study drug by the investigator. For laboratory results, only a number of explicitly defined laboratory adverse events were considered to be DLT (details can be found in the Supplementary Table 2). If two or more patients reached DLT in one dose level, maximum tolerated dose (MTD) was defined at the last dose level beneath DLT. A maximum of five dose levels were originally planned ranging from a single dose of 10 mg to four times a weekly dose of 40 mg.

### Primary and secondary endpoints

To assess safety as a primary outcome, patients were evaluated for presence of and changes in adverse events during each visit and were asked to report symptoms if those had occurred between visits. Adverse events (AEs) and serious adverse events (SAE) were registered and graded in accordance with the National Cancer Institute Common Terminology Criteria for AEs (CTCAE Version 4.03). On pre-defined time points around each dosing, safety laboratory assessments were done (blood chemistry, urine analysis, and hematology). The full schedule of assessments can be found in Supplementary Table 1. Preliminary efficacy was a secondary endpoint and was assessed as response according to IMWG Criteria [27] at week 4 and 8 after the first dose in serum (protein electrophoresis (M-gradient), involved immunoglobulin heavy chain, free kappa, and lambda light chains) and in urine (free kappa and lambda light chains). For assessment of quality of life (QoL), the NCCN distress thermometer as well as a pain visual analog scale (VAS) were used. For pharmacokinetic evaluation, blood samples were collected at day 0 (before treatment administration, immediately after the end of the infusion at *t* = 0, and 2 h after end of infusion), as well as on days 1, 3, 7, 14, 21, 28, 42, and 56. In these samples, both liposomal dexamethasone sodium phosphate and free (released) dexamethasone were assessed, taking into account the fact that phosphatases extremely efficiently convert the phosphate form in plasma into the parent drug and thus all dexamethasone phosphate is liposomal while free dexamethasone represents the released drug [28].

### Statistical analysis

Data listings were provided for PK and safety data. Summaries were presented overall and by cohort. Summary statistics for continuous data contained number of subjects, mean, median, standard deviation, and range (minimum, maximum); summary statistics for categorical data contained number and percentage. The Safety Population includes all subjects who received all or part of the study drug who have at least one post-dose safety assessment. The pharmacokinetic population (PP) includes all subjects who received all or part of the study drug and have at least one valid PK parameter.

## Results

### Patient characteristics

In total, seven patients were enrolled in this study and all gave informed consent. Due to rising number of competing trials and increasing number of successfully proven new treatment alternatives during the time of study initiation, enrollment had to be prematurely stopped after two dose levels due to slow recruitment. One patient in the second dose level did not reach the week 8 assessment due to progressive disease with development of a plasma cell leukemia, leading to the recruitment of an additional patient. All patients had progressive multiple myeloma according to IMWG criteria, but no immediate clinical need of a line of treatment. Of the 7 patients, 4 were male and 3 were female. At baseline, the median age was 81 (range 52–87 years) and the mean weight was 70 kg (range 57–91 kg). Further details are given in Table 1.

**Table 1:** Characteristics of patients enrolled in the study

	**Dose level 10 mg** (*n* = 3)	**Dose level 40 mg** (*n* = 4)
Median age (years)	81 (range 52–86)	75.5 (range 52–87)
Male patients	66.7%	50%
Median height (cm)	172 (range: 163–178)	166 (range: 162–170)
Median weight (kg)	65 (range: 63–91)	68 (range: 57–80)
Median number of concomitant diseases	3 (range: 2–5)	3 (range: 1–4)
Median number of previous lines of chemotherapy	3 (range: 2–4)	4 (range: 2–6)
Median number of concomitant medications	7 (range: 4–8)	3 (range 2–4)
Median M-protein at baseline (g/dl)	1.6 (range: 1.05–2.1)	1.5 (range: 0.1–2.0)
Median creatinine level at baseline (mg/dl)	1.32 (range: 0.81–1.56)	1.20 (range: 0.81–1.73)
Median baseline leukocytes (count/nl)	3.7 (range: 2.1–11.8)	7.5 (range: 4.3–10.1)
Median baseline erythrocytes (count/pl)	3.6 (range: 3.3–4.2)	3.7 (range: 3.0–4.0)
Median baseline platelets (count/nl)	249 (range: 117–326)	121 (range: 62–185)

### Safety evaluation

The safety results are summarized in Table 2. The occurrence and severity of adverse events (AEs), the occurrence of systemic AEs as measured by potentially clinically significant changes in ECG, vital signs, physical examinations, and laboratory tests, as well as the occurrence of injection site reactions were part of the main endpoints. AEs were graded in line with the Common Terminology Criteria for Adverse Events (CTCAE) version 4.03 (https://ctep.cancer.gov/protocoldevelopment/electronic_applications/ctc.htm).

**Table 2:**
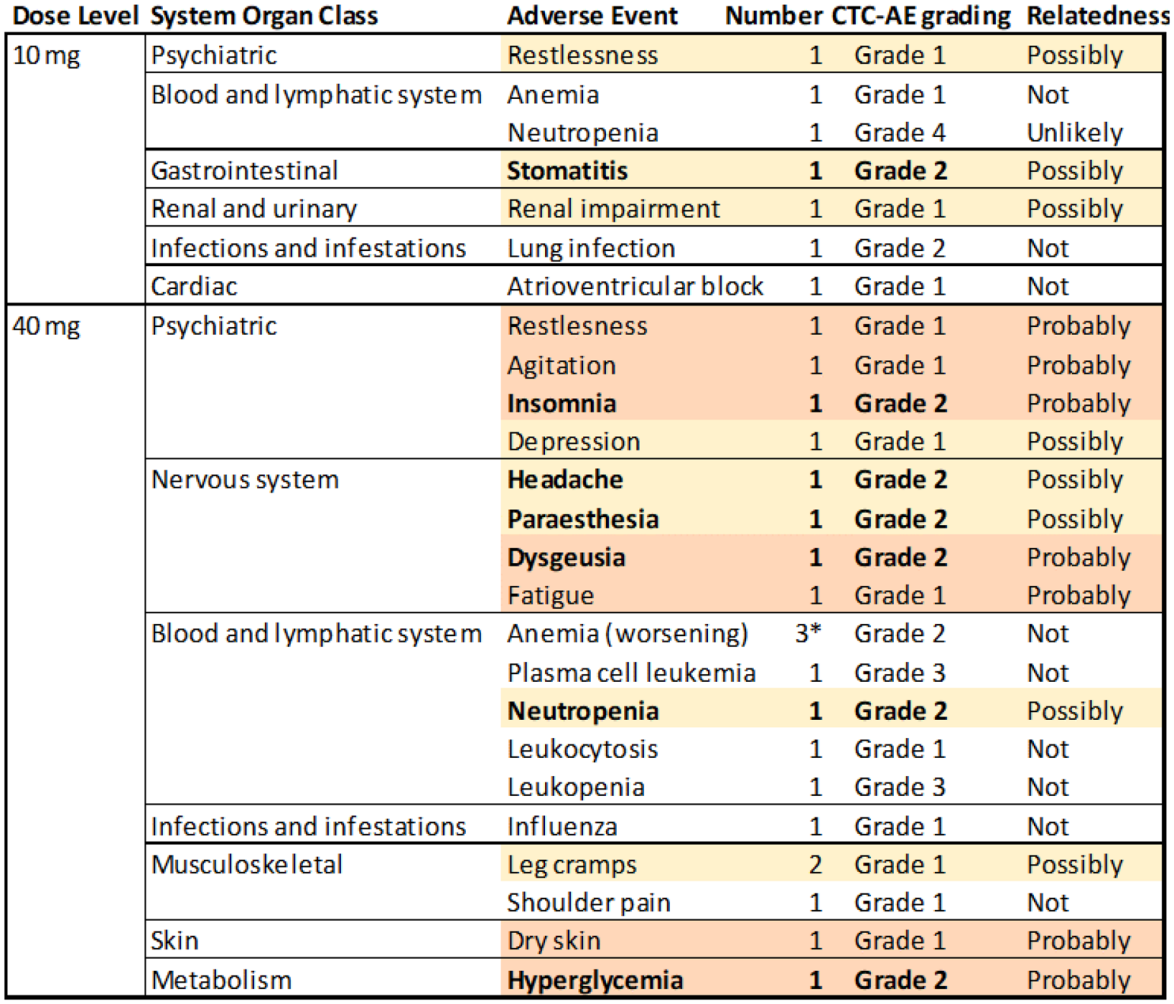
Adverse events (AEs) observed in the patients, graded for relatedness and severity. Light amber shaded lines indicate “possibly related”, light orange “probably related” as rated by the investigator, with related Grade 2 adverse events shown in bold

* Indicates 3 identical eventsobserved consecutively in the same person. It needs to be mentioned that thepsychiatric and nervous system-related AEs in this table were observed in onesingle patient

There were no deaths reported in this study. In total 28 AEs were reported, and they were seen in all 7 patients. Eleven out of 28 AEs in total were categorized as ‘not related’ to the study drug, one was rated ‘unlikely related’ while 8 were judged ‘possibly related’ and 7 ‘probably related’. The most frequent AEs fell in the category of blood and lymphatic system disorders, with 9 events recorded in 5 out of 7 patients. In general, no apparent clinically significant changes or abnormalities were observed during the study period, which would have led to termination of the study. No infusion reactions were observed. One patient experienced a neutropenia that was graded of life-threatening severity. This subject had a long-standing fluctuating leuko- and neutropenia, due to impaired bone marrow function as a result of pre-treatment. It was, therefore, judged to be unlikely related to the study drug. The leuko- and neutropenia were not accompanied by clinical symptoms and lasted only one day. Knowing that i.v. administered liposome products tend to be taken up by liver macrophages [18], it is relevant to note that no liver toxicity was seen in these patients.

No dose-limiting toxicities occurred in both dose levels. There were no serious adverse events (SAEs) and no hospitalizations due to AEs. In the second cohort, one patient suffered from premature progression of disease (development of plasma cell leukemia) and could not be kept in the study cohort until week 8. Therefore, an additional patient was added to this cohort.

### Pharmacokinetics

**Fig. 2:**
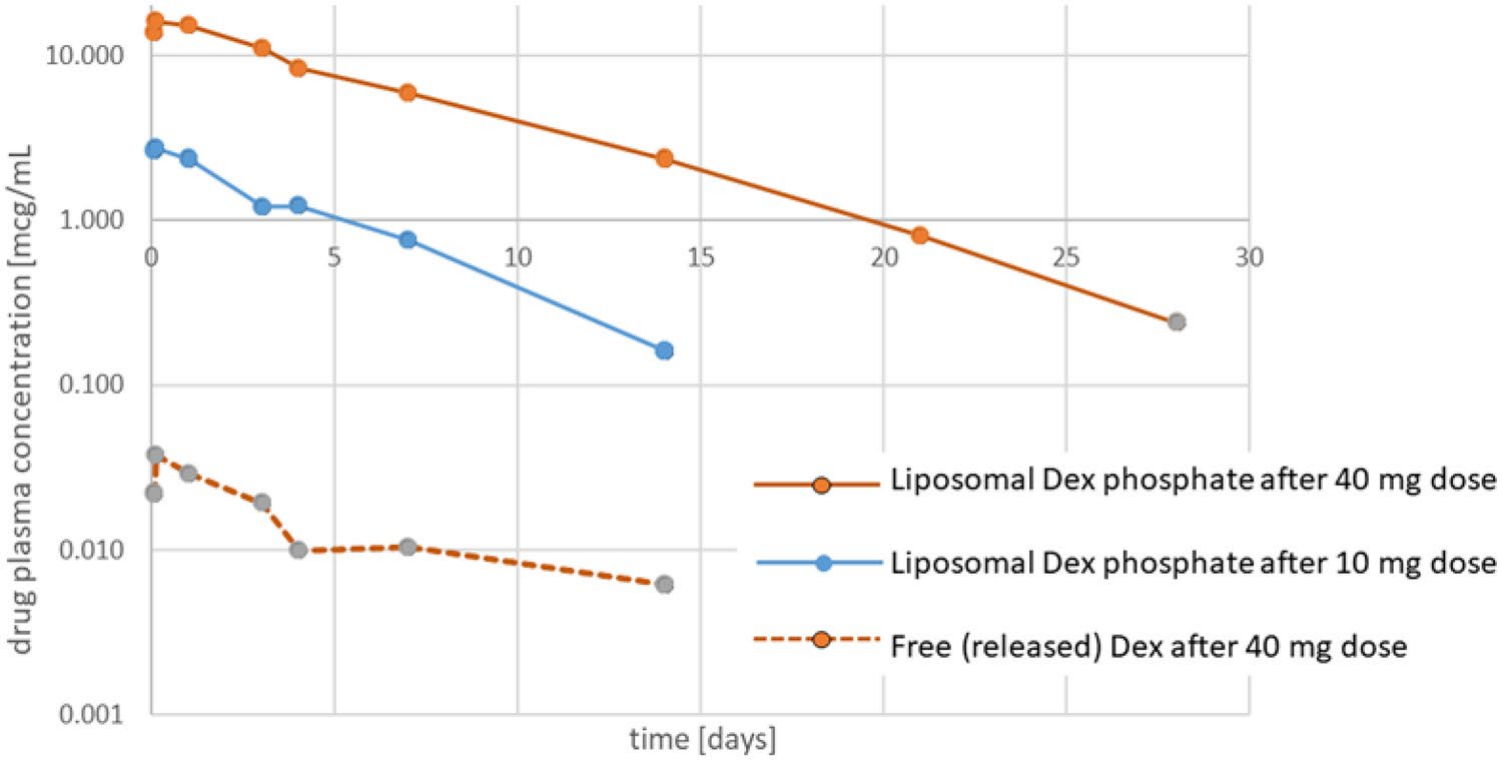
Pharmacokinetic profiles of encapsulated dexamethasone phosphate and free dexamethasone measured simultaneously upon i.v. infusion of two different doses of PEG-liposomal dexamethasone

While a complete picture of the pharmacokinetic behavior of PEG-liposomal dexamethasone could not be obtained with only limited data from 7 patients, the results shown in Fig. 2 confirm observations with PEG-liposomal drug products reported by others [29]. In line with those publications, the very high plasma levels and long circulation half-lives observed with liposomal dexamethasone are striking. They indicate that the volume of distribution of the liposomes is not much larger than the plasma volume itself.

The curves also show that with the 40 mg liposomal dexamethasone dose some free dexamethasone enters the circulation upon liposome administration, presumably as a result of liposomal clearance by liver macrophages and lymphoid organs, knowing that the liposomes are stable and are not prematurely leaking drug in the circulation [28]. However, compared to the encapsulated dexamethasone phosphate concentrations, the systemic exposure to free dexamethasone is proportionally very low, and most marked only in the first week after liposome administration. In Table 3, the calculated pharmacokinetic parameters for liposomal dexamethasone at the two dose levels are shown and compared to literature data obtained with i.v. and oral free dexamethasone.

**Table 3:** Pharmacokinetic parameters of liposomal dexamethasone phosphate vs. free dexamethasone at different dosing levels upon i.v. infusion

	i.v. liposomal Dex 10 mg	i.v. liposomal Dex 40 mg	i.v. free Dex 150 mg* [30]	i.v. free Dex 4 mg* [31]	Oral free Dex 6 mg* [31]	Unit
Plasma conc at *t* = 0 (*s*_0_)	2.7	16.4	3.6	0.1	0.068 (Cmax)	mcg/mL
Distribution volume (*V*_*d*_)	0.050	0.035	1.0	0.94	1.09	L/kg
Plasma half-life (t_1/2_)	83.4	113.4	4.0	9.0	6.9	hrs
Clearance (CL)	0.031	0.015	11.6	6.4	7.7	L/hrs
Area under the curve (AUC)	326,136	2,685,826	114	626	774	mcg*hrs/L

* Indicates free dexamethasone data obtained from literature

### Preliminary efficacy results

As only two dose levels could be fully recruited and therefore no patient received more than one dose of the drug, efficacy results are limited. Clinical parameters are indicating that the activity of MM stayed stable in the 40 mg dose group during the 8 weeks of assessment while it tended to rise in the 10 mg treatment group: mean M-protein levels were 1.26 g/dl at baseline in the 40 mg cohort and 1.15 g/dl at week 8 after treatment, while in the 10 mg cohort, the baseline level was 1.58 g/dl and climbed to 2.91 g/dl at week 8.

**Fig. 3:**
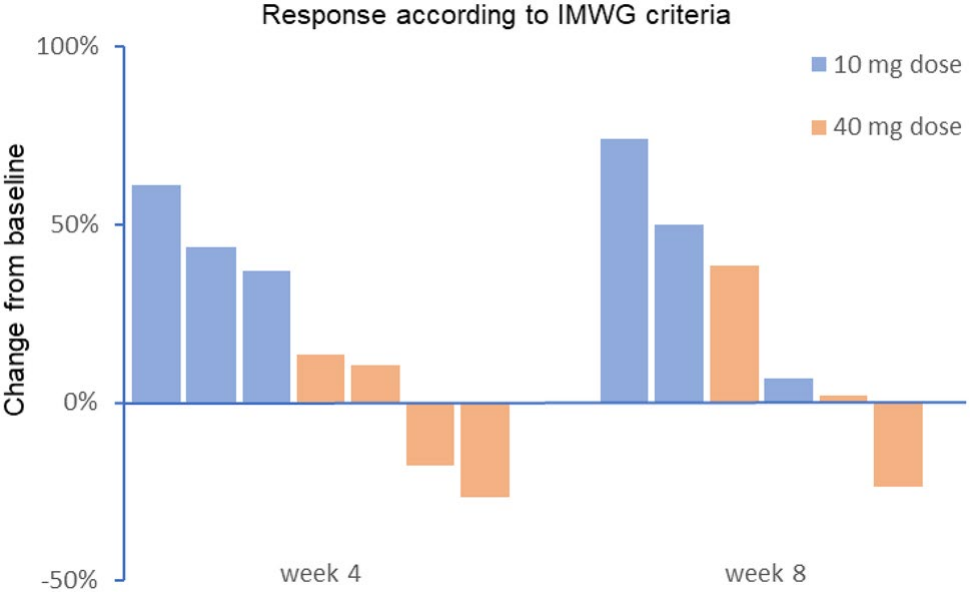
IMWG response calculated as percentage change from baseline of serum levels of immunoglobulin heavy chain or—in case of light chain myeloma—the free light chain, at week 4 and 8 after the i.v. infusion of PEG-liposomal dexamethasone phosphate. Bars show individual patients. Blue are patients in the 10 mg and orange in the 40 mg dose group

This picture was confirmed by the immunoglobulin assessments. Figure 3 shows changes in the serum concentrations of immunoglobulin heavy chain or—if the patient that had light chain MM—free kappa and lambda light chains over the 8 weeks of assessment. With progression defined as more than 25% increase in these serum levels, stable disease as between 25% increase and 50% reduction, and a therapeutic response as more than 50% reduction, all patients in the 10 mg dose group showed disease progression, while all patients in the 40 mg group showed stable disease. At week 8, the picture is less clear with stable disease shown with 1 out of 3 patients in the 10 mg group and in 2 out of 3 patients in the 40 mg group (one patient in the 40 mg group was missing due to premature disease progression).

## Discussion

We conducted a combined phase I/IIa open label interventional trial designed to evaluate the safety and efficacy of a pegylated liposome formulation of dexamethasone (Dex-PL). We found that a single intravenous infusion of liposomal dexamethasone was well tolerated, both during the infusion and the follow-up study period. Regarding pharmacokinetics, while the type of patients treated IV with Dex-PL was clearly different from the patients in the referenced study on free dexamethasone, the multifold lower Cl and Vd with liposomal dexamethasone are hard to miss, as is the more than tenfold longer half-life (Table 3). Indeed, liposomal encapsulation seems to increase the AUC of the drug with more than a factor 200, taking into account the difference in dose level.

The increasing number of treatment options developed during the past decade have turned MM from a cancer with a poor survival outcome into a more chronic disease mostly treated in an outpatient setting in which clinically quieter stages (e.g., under oral maintenance treatment) alternate with relapses that require changes in the (now mostly targeted) treatment strategy. Almost all conventional drug therapies in MM are combinations in which dexamethasone represents an important backbone. Also, in the new targeted combination treatment approaches that involve proteasome inhibitors, immunomodulatory drugs, and/or monoclonal antibodies, dexamethasone retains its place as standard-of-care [32]. The strong immunosuppressive activity of glucocorticoids (GC) and in particular dexamethasone are thought to amplify the immunomodulatory activity of these more targeted drugs. On the other hand, however, GC are characterized by a significant side effect profile that includes a substantially increased chance of infections particularly in combination with immune-modulatory drugs in MM [33]. Considering the fact that infections are one of the main risks in the long-term management of MM especially during chemotherapeutic regimens, the search for new effective MM drugs has also been a quest to reduce infection-related morbidity and mortality.

Outside the field of cancer, several lines of investigation have been pursued to improve the therapeutic index of systemically administered GC [34], notable examples being the more selective glucocorticoid receptor agonists. Another widely explored strategy resides in the selective delivery of GC to pathological sites using nanomedicines, with several GC nanodrugs currently in (pre)clinical development [35] and with a good amount of preclinical proof-of-concept available in experimental tumor models [36–38].

The most advanced GC nanodrug is pegylated liposomal prednisolone, which was recently shown to improve efficacy over GC standard-of-care in a large cohort of patients with active rheumatoid arthritis [39]. The same product was also evaluated in patients with severe orbital inflammation due to Graves’ disease and in patients on dialysis with arteriovenous fistula failure [40, 41]. The use of the same liposome system encapsulating the 6.5-fold more potent dexamethasone, and thus theoretically requiring less liposomes for the same effect, seems a logical next step especially for indications in which dexamethasone is the clinically preferred GC. Indeed, dexamethasone tends to be the GC of choice in several types of cancer, of which MM is the disease in which there is the highest unmet medical need for improving the efficacy-safety ratio of the drug. Recently, a small clinical trial was completed with PEG-liposomal dexamethasone in cancer patients with castrate-resistant prostate carcinoma in which the safety of repeated doses of up to 18.5 mg was assessed [42]. This was based on preclinical studies with liposomal dexamethasone revealing that there may be additional therapeutic benefit of enhanced local delivery to tumor sites. First, the drug may impact the extracellular matrix density in and around tumors, thereby enhancing drug delivery to and into tumors [43]. Furthermore, liposomal dexamethasone has been investigated in preclinical models of prostate cancer, where it appears to block the growth of bone metastases, and does so more effectively than free dexamethasone [44].

Although the current dataset is limited, we believe the results presented here support the hypothesis that long-circulating liposomes are safe and (pending confirmation in follow up trials) may provide for a favorable tolerability of dexamethasone therapy in MM. Among the most interesting findings are the changes in pharmacokinetic behavior of the GC conferred by liposomal encapsulation. Interestingly, the enormously increased area under the curve (AUC) does not seem translate in high systemic activity, suggested by the limited number of adverse effects observed. Possible steroid-related psychiatric and nervous system-related side effects were seen in one patient. These side effects mostly resolved in the weeks after treatment. With regard to infections, it is noteworthy that although a moderate lung infection and a mild influenza were reported, these resolved and were judged as not related by the investigator.

The observation that a multifold larger AUC does not lead to marked and substantial systemic dexamethasone effects corroborates the assumption that dexamethasone largely remains in its inactive phosphate prodrug form, safely encapsulated in the liposomes, limiting systemic exposure. At the same time, the high plasma concentrations may result in high drug concentrations at target sites, because MM bone marrow lesions are characterized by increased micro-vessel density, permeability, and by ongoing angiogenesis, which could render the target tissue directly accessible to the liposomes [43–45]. Here, populations of phagocytosing immune cells can take up and digest the liposomes, liberating the dexamethasone phosphate prodrug and converting it into active dexamethasone that can either exert its pharmacological activity in these immune cells or upon release in other key cells in the pathological lesions, including the myeloma plasma cells.

An obvious limitation of this study is the small number of patients due to the premature closing of the trial. The study protocol intended a higher number of patients with higher and also repeated doses of dexamethasone. Unfortunately, recruitment into the study became increasingly difficult. When the study was planned, only lenalidomide and bortezomib were widely available. Finally, when the study was running, several more and well-tolerated drugs had been approved for the treatment of multiple myeloma, making inclusion into a study with dexamethasone monotherapy difficult.

The administration of liposomal dexamethasone up to a single dose of 40 mg seems safe and led to extended circulating drug levels, and no dose-limiting toxicities were observed yet. The levels of circulating free active drug were low. There appeared to be a dose-dependent pharmacological effect, as 40 mg of Dex-PL lead to disease stabilization in 4 out of 4 patients at week 4, while 10 mg of Dex-PL resulted in progressive disease in 3 out of 3 patients. Further studies are needed to investigate higher doses, to characterize side effects as well as to identify of the dose-limiting toxicity, and above all to obtain evidence for improved efficacy. Possibly, liposomal dexamethasone can serve as a combination formulation in many drugs regimens in the future, with higher drug concentrations in the bone marrow and fewer side effects due to lower systemic exposure. All in all, this first-in-man MM patient study may provide a stepping stone to future studies in which this potentially safer form of dexamethasone is explored alongside other and newer MM drug combinations.

## Supplementary Information

Below is the link to the electronic supplementary material.

Supplementary file1 (PDF 94 KB)

## Data Availability

The datasets generated during the current study are available from the corresponding author on reasonable request.
