# Supplementary material for: A phase I first-in-man study to investigate the pharmacokinetics and safety of liposomal dexamethasone in patients with progressive multiple myeloma
Source: Drug Deliv Transl Res. 2023 Jan 2;13(4):915–23. doi: 10.1007/s13346-022-01268-6 (PMC9981510; doi:10.1007/s13346-022-01268-6)
Supplement: Supplementary file 1 — Supplementary file1 (PDF 94 KB) [file 13346_2022_1268_MOESM1_ESM.pdf]

Metselaar et al., Liposomal dexamethasone in multiple myeloma

Supplementary Information

Table 1. Schedule of Assessments

Study Procedure	Screening (Day -14 to -1)	Baseline (Day 0)	Treatment (Week 1, Day 7±1)	Treatment (Week 2, Day 14±1)	Treatment (Week 3, Day 21±2)	Safety Visit (Week 4, Day 28±2)	Safety Visit (Week 6, Day 42±3)	End of Study Visit (Week 8, Day 56±3)
Informed consent	X							
Inclusion/exclusion criteria	X							
Medical history	X							
Demography	X							
Vital signs:<br>body temperature, respiratory rate, blood pressure, heart rate, height and weight	X	X	X	X	X	X	X	X
Physical examination, Karnowski PS	X	X	X	X	X	X	X	X
Adverse events evaluation		X	X	X	X	X	X	X
Concomitant medication	X	X	X	X	X	X	X	X
Quality of life (QoL) assessment		X				X		X
Standard laboratory evaluation	X	X	X	X	X	X	X	X
Extended laboratory evaluation	(X)	X				X	X	X
Myeloma assessment<br>Serum: Protein electrophoresis (M-gradient), involved Ig heavy chain, free kappa and lambda light chains<br>24h-Urine: free kappa and lambda light chains[3]		X				X		X
Serum morning cortisol		X	X			X	X	
Serum pregnancy test	X							
12-lead electrocardiogram (ECG)	X					X	X	
**Administration of Dex-PL**		**X**	**(X)**	**(X)**	**(X)**			
PK Sampling (additionally Day 1 and Day 3)		X	X	X	X	X	X	X
Biomarker sampling		X						

Table 2. Laboratory events that are considered dose limiting toxicities (DLT) (if judged to be related to the study drug by the investigator).

	DLT when:
White blood cells	<1.0 G/L
Absolute neutrophil counts	<1.0 G/L
Platelets	<25 G/L
Sodium	<125 mmol/L<br>>155 mmol/L
Potassium	<2.5 mmol/L<br>>6.0 mmol/L
Calcium	<1.75 mmol/L<br>>3.4 mmol/L
Chloride	N/A
Phosphorous	<0.6 mmol/L
Total bilirubin	>3.0x Upper Limit of Normal (ULN)
Alkaline phosphatase	>5x ULN
Gamma-glutamyl transferase	>5x ULN
Alanine aminotransferease	>5x ULN
Lactate dehydrogenase	N/A
Creatine kinase	>5x ULN
Creatinine	>3x ULN or >3x baseline
Fasting glucose	>500 mg/dL
Albumin	<2 g/dL
Cholesterol	>400 mg/dL
